# First insights on value-based healthcare of elders using ICHOM older person standard set reporting

**DOI:** 10.1186/s12877-020-01734-1

**Published:** 2020-09-09

**Authors:** Wei-Ju Lee, Li-Ning Peng, Chi-Hung Lin, Shinn-Zong Lin, Ching-Hui Loh, Sheng-Lun Kao, Tzu-Shing Hung, Chia-Yun Chang, Chun-Feng Huang, Ting-Ching Tang, Liang-Kung Chen

**Affiliations:** 1 Aging and Health Research Center, National Yang Ming University, Taipei, Taiwan; 2 Department of Geriatric Medicine, School of Medicine, National Yang Ming University, Taipei, Taiwan; 3 Department of Family Medicine, Taipei Veterans General Hospital Yuanshan Branch, Yi-Lan County, Taiwan; 4 Center for Geriatrics and Gerontology, Taipei Veterans General Hospital, No. 201, Sec. 2, Shih-Pai Rd, Taipei, 11217 Taiwan; 5 Superintendent’s Office, Hualien Tzu Chi Hospital Buddhist Tzu Chi Medical Foundation, Hualien County, Taiwan; 6 Center of Health and Aging, Hualien Tzu Chi Hospital Buddhist Tzu Chi Medical Foundation, Hualien County, Taiwan; 7 Yingge Primary Care Center, Department of Health, New Taipei City Government, New Taipei City, Taiwan; 8 Shulin Primary Care Center, Department of Health, New Taipei City Government, New Taipei City, Taiwan; 9 Department of Family Medicine, National Yang-Ming university Hospital, Yi-Lan County, Taiwan; 10 Tang’s Orthopedic & Otolaryngological Clinic’s, New Taipei City, Taiwan

**Keywords:** International consortium for health outcomes measurement, Elder adult, Age, Value, Healthcare

## Abstract

**Background:**

Clinical guidelines for specific conditions fragment care provision for elders. The International Consortium for Health Outcomes Measurement (ICHOM) has developed a global standard set of outcome measures for comprehensive assessment of older persons. The goal of this study was to report value-based health metrics in Taiwan using this ICHOM toolset.

**Methods:**

The cross-sectional study of baseline data excerpted from a prospective longitudinal cohort, which recruited people ≥65 years old with ≥3 chronic medical conditions between July and December 2018. All participants received measurements of physical performance, anthropometric characteristics, health-related behaviors, Charlson Comorbidity Index, and Montreal Cognitive Assessment. The ICHOM toolset comprises three tiers: 1 includes frailty and having chosen a preferred place of death; 2 includes polypharmacy, falls, and participation in decision-making; and 3 includes loneliness, activities of daily living, pain, depression, and walking speed. These items were converted into a 0–10 point value-based healthcare score, with high value-based health status defined as ≥8/10 points.

**Results:**

Frequencies of individual ICHOM indicators were: frail 11.7%, chose preferred place of death 14.4%, polypharmacy 31.5%, fell 17.1%, participated in decision-making 81.6%, loneliness 26.8%, limited activities of daily living 22.4%, pain 10.4%, depressed mood 13.0%, and slowness 38.5%. People with high disease burden (OR 0.40, 95% CI 0.21–0.76, *p* = 0.005) or cognitive impairment (OR 0.49, 95%CI 0.27–0.87, *p* = 0.014) were less likely to have high value-based healthcare status.

**Conclusions:**

The ICHOM Standard Set Older Person health outcome measures provide an opportunity to shift from a disease-centric medical paradigm to whole person-focused goals. This study identified advanced age, chronic disease burden and cognitive impairment as important barriers to achieving high value-based healthcare status.

## Background

A single-disease model has prevailed over centuries of medical progress, but the era of population aging brings major challenges of managing multimorbidity in older adults that threaten to fragment care provision by necessitating multiple assessments and treatments [1]. Healthcare systems will be increasingly burdened by fragmented services, higher service volumes and escalating associated medical costs, and are hence transitioning from volume-based to value-based provision that emphasizes quality, expenditure and patient experience [2]. Consequently, the question of how best to measure healthcare quality and outcomes has become a research priority. Specific models of value-based healthcare, such as pay-for-performance, have shown effectiveness in certain diseases or chronic conditions but not overall [3]. Moreover, prevalent chronic comorbidities in older adults [1] make it hard to measure variations in health outcomes. More ‘function-centric’ aging medicine is crucial to handling the diversity and complexity of health care for older people and promoting healthy aging [4].

The International Consortium for Health Outcomes Measurement (ICHOM) has initiated an ambitious project to develop value-based health metrics for specific groups of people rather than discrete diseases/conditions. To establish a standard health outcome set and improve care and quality pathways for older adults, the ICHOM convened a global expert consensus panel to formulate evidence-based outcome measurement tools [5]. Without such tools, it is difficult for policymakers and health professionals to choose interventions effective in improving care quality [6]. The ICHOM Standard Set of health outcomes for older persons will be conductive to supplanting piecemeal care of older persons with a more holistic approach. ICHOM standard set for older adults might provide an operative definition for high value-based healthcare services and an opportunity for healthcare providers and policy makers to examine and refine services provisions.

Since the ICHOM Standard Set Older Person was published in 2018, important questions remain. For example, which patient subgroups require comprehensive assessments to evaluate their needs? Reporting these health metrics is the first step towards pragmatic application of this tool. This study explored the application of the ICHOM Standard Set for Older Persons health status among older multi-morbid community-dwelling adults in Taiwan.

## Methods

### Participants and study design

This cross-sectional study recruited older multimorbid community-living adults in New Taipei City, Yi-Lan County, and Hualien County, Taiwan, between July and December 2018. The inclusion criteria were: age ≥ 65 years and ≥ 3 chronic medical conditions. The study excluded people who: were unable to communicate adequately with study staff; had malignant tumors undergoing active chemotherapy; with life expectancy < 12 months; were institutionalized. Supplementary figure 1 showed details of recruitment process.

This study was designed and conducted in accordance with the principles of the Declaration of Helsinki. The Institutional Review Board of National Yang-Ming University approved the protocol (YM107042F). All participants provided fully informed written consent. The design and reporting format follow STROBE guidelines [7].

### Value-based health metrics

The ICHOM Standard Set Older Person comprises three tiers (Supplementary Table 1) [5]. Absent valid Taiwanese versions of 4-item screening Zarit Burden interview- measured carer burden- and the Adult Social Care Outcomes Toolkit -measured autonomy and control- mitigated their applications in this study. Mortality was excluded because of present baseline data. Tier 1, achieved or retained health status, includes: all cause survival; death in a chosen place; and frailty. Participants were asked whether they had expressed a preferred place to die, and frailty was defined as clinical frailty scale ≥4 [8].

Tier 2, treatment burden and complications, includes: falls in the last 12 months; polypharmacy with ≥5 drugs [9]; and participation in decision-making, which comprised confidence in ability to manage their own health, discussion and planning of care, being treated with dignity and respect, coordination of care, and discharge to a chosen place. People in whom of these all components were affirmed were classed as having high participation in decision-making.

Tier 3, long-term consequences of care management and health sustainability, includes: loneliness, defined as ≥35 points on the University of California, Los Angeles (UCLA) loneliness scale [10, 11]; limitation of daily activities (disability), defined as Lawton instrumental activities of daily living scale < 8 (most independent) [12]; 6-m walk speed at usual pace, with < 0.8 m/s defined as slowness [13]; pain and emotional health measured by the Short-Form Health Survey (SF-36), with pain affecting activities of daily living considered pain, and the criterion for depression being ≥5/9 SF-36 depressive symptoms [14].

Based on items in Tiers 1, 2 and 3 (Supplementary Table 1), a score ranging from 0 to 10 was derived to represent the value-based health status of each individual; a highest tertile score of ≥8/10 was defined as high value-based health status.

### Other variables

Physical performance, anthropometric characteristics, and health-related behaviors of all participants were recorded. Any tobacco or alcohol use in the last 6 months was classed as smoking or drinking, respectively. Exercise was defined as fitness activity for ≥30 min at least thrice weekly. Blood pressure, height and body weight were measured by standard procedures; body mass index was calculated as weight in kilograms, divided by height in meters squared (kg/m^2^). All participants were asked whether they had signed a Do Not Resuscitate order, which is an official agreement registered on national health insurance cards. Cognitive function was measured using The Montreal Cognitive Assessment (MoCA), adjusted by adding one point for those educated for ≤12 years (MoCA_adj_); MoCA_adj_ ≥ 26 constituted normal cognitive function [15]. Charlson Comorbidity Index quantified disease burden and comorbidity burdens, with high burden defined as a score of ≥2 [16].

### Statistical analysis

All analyses were performed with the SAS statistical package, version 9.4 (SAS Institute, Inc., Cary, NC, USA). A two-sided *p*-value < 0.05 was considered statistically significant. Numerical variables were expressed as mean plus/minus standard deviation and categorical variables as proportions. Descriptive characteristics were compared by Student t test or chi-square analysis, as appropriate. To maximize statistical efficiency, the value-based healthcare score was first treated as a continuous variable, then univariable and multivariable logistic regression analyses were used to investigate associations between corresponding variables and higher value-based healthcare status; *p* < 0.1 in univariable analysis was the entry criterion for multivariable analysis.

## Results

### Participant characteristics

**Fig. 1:**
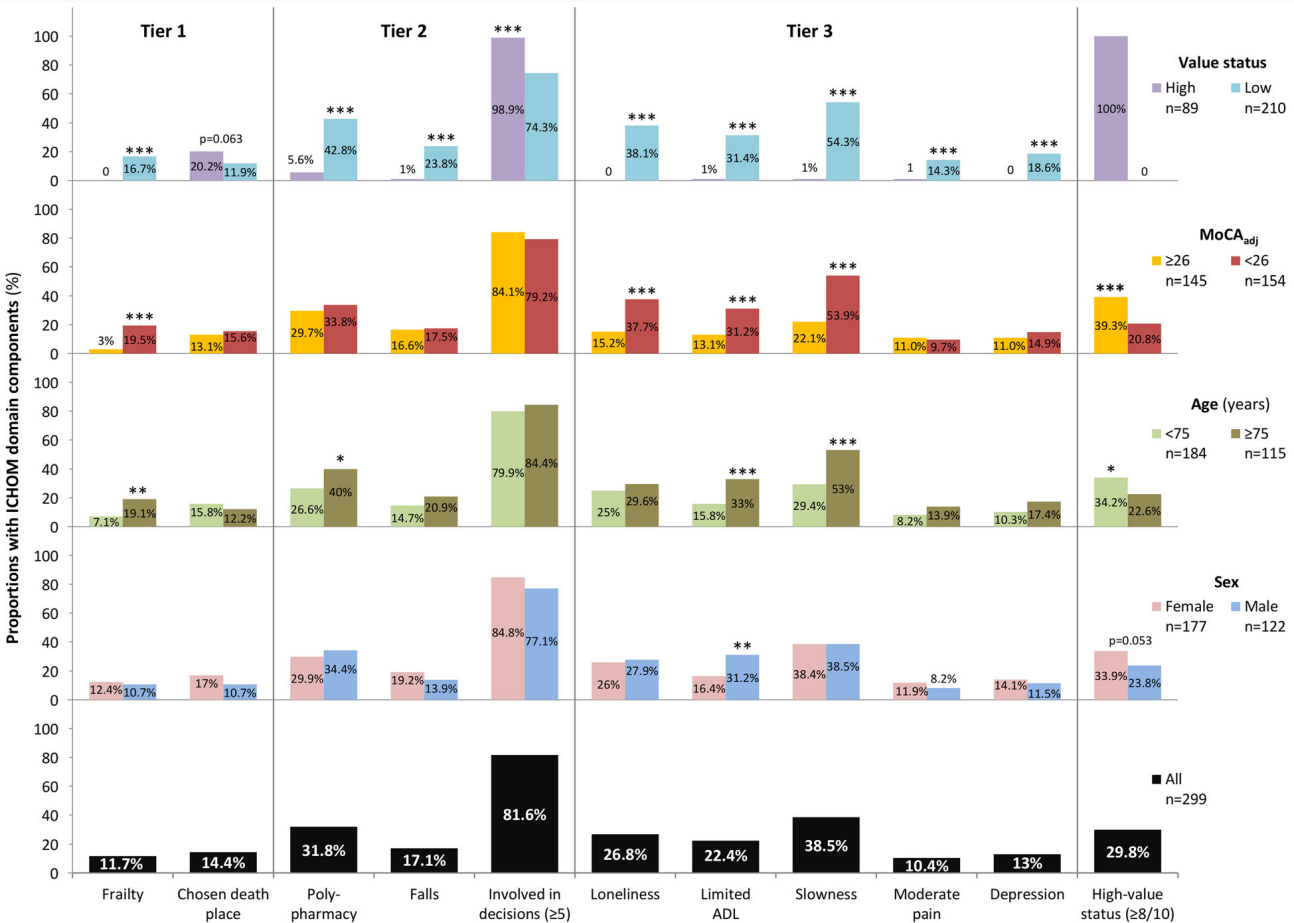
Comparison of individual ICHOM Tiers and total value-based health care score by value-based health status, age, sex, and cognitive performance. ICHOM, International Consortium for Health Outcomes Measurement; ADL, activities of daily living; MoCA_adj_, Montreal Cognitive Assessment adjusted (one point added for education years ≤12)

The mean value-based healthcare score of 299 enrolled participants was 7.2 ± 1.8 and 89 (29.8%) had high value-based healthcare status (Fig. 1, Table 1). Although all participants had three or more chronic conditions, the mean Charlson Comorbidity Index score was only 1.1 ± 1.0 (Table 2). A minority of participants had chosen a place to die and signed Do Not Resuscitate agreements, but more than 90% reported a high level of participation in care plan decision-making. One-quarter experienced moderate loneliness and one in eight had depressed mood (Table 1).

**Table 1:** ICHOM Standard Set Older Person outcome measures by value-based health status

Data values show mean ± standard deviation or number (percent)	Entire cohort	Value-based health status		***p***
		Low (< 8/10)	High (≥8/10)	
Number	299	210	89	
**ICHOM Standard Set Older Person Tier 1**				
Clinical frailty scale	2.7 ± 0.9	2.8 ± 1.0	2.3 ± 0.7	< 0.001
Frail	35 (11.7)	35 (16.7)	0 (0.0)	< 0.001
Preferred place of death chosen	43 (14.4)	25 (11.9)	18 (20.2)	0.061
Do Not Resuscitate signed	21 (7.0)	11 (5.2)	10 (11.2)	0.064
**ICHOM Standard Set Older Person Tier 2**				
Number of drugs	3.6 ± 2.7	4.3 ± 2.7	2.0 ± 1.7	< 0.001
Polypharmacy (≥5 concurrent drugs)	95 (31.8)	90 (42.8)	5 (5.6)	< 0.001
Number of adverse drug events in past 12 months	0.0 ± 0.1	0.0 ± 0.2	0.0 ± 0.0	0.180
Episodes of discomfort after medications in past 12 months	0.0 ± 0.1	0.0 ± 0.2	0.0 ± 0.0	0.103
Fell in past 12 months	51 (17.1)	50 (23.8)	1 (1.1)	< 0.001
Number of falls in past 12 months	0.2 ± 0.6	0.3 ± 0.7	0.0 ± 0.1	< 0.001
Hospital admissions in past 12 months	0.2 ± 0.4	0.2 ± 0.5	0.1 ± 0.3	0.004
Length of hospital stay (days)	1.1 ± 3.7	1.4 ± 4.3	0.5 ± 1.8	0.009
Able to cope with own health	276 (92.3)	187 (89.1)	89 (100.0)	0.001
Participate in care decision-making	276 (92.3)	188 (89.5)	88 (98.9)	0.006
Treated with dignity and respect	289 (96.7)	200 (95.2)	89 (100.0)	0.036
Received coordinated care	270 (90.3)	181 (86.2)	89 (100.0)	< 0.001
Discharged to place of choice	296 (99.0)	207 (98.6)	89 (100.0)	0.257
Overall participation in decision-making	4.7 ± 0.7	4.6 ± 0.9	5.0 ± 0.1	< 0.001
High participation (≥5)	244 (81.6)	156 (74.3)	88 (98.9)	< 0.001
**ICHOM Standard Set Older Person Tier 3**				
UCLA Loneliness Scale	31.0 ± 10.0	33.4 ± 11.0	25.3 ± 3.0	< 0.001
Loneliness	80 (26.8)	80 (38.1)	0 (0.0)	< 0.001
Activities of daily living	7.4 ± 1.4	7.2 ± 1.6	8.0 ± 0.3	< 0.001
Any limitation of activities of daily living	67 (22.4)	66 (31.4)	1 (1.1)	< 0.001
Walking speed (m/s)	0.9 ± 0.3	0.8 ± 0.3	1.1 ± 0.2	< 0.001
Slowness (6-m walk < 0.8 m/s)	115 (38.5)	114 (54.3)	1 (1.1)	< 0.001
Moderate pain	31 (10.4)	30 (14.3)	1 (1.1)	< 0.001
Depression	39 (13.0)	39 (18.6)	0 (0.0)	< 0.001
Value-based healthcare score	7.2 ± 1.8	6.5 ± 1.6	9.1 ± 0.3	< 0.001
High value-based healthcare	89 (29.8)	0	89 (100)	< 0.001

*ICHOM* International Consortium for Health Outcomes Measurement, *UCLA* University of California, Los Angeles

**Table 2:** Demographic and health-related characteristics by value-based health status

Data values show mean ± standard deviation or number (percent)	Entire cohort	Value-based health status		***p***
		Low (< 8/10)	High (≥8/10)	
**Demographics and health-related factors**				
Number	299	210	89	
Age (years)	73.3 ± 6.6	74.0 ± 6.9	71.5 ± 5.7	0.002
Male	122 (40.8)	93 (44.3)	29 (32.6)	0.060
Education (years)	7.6 ± 4.7	7.3 ± 4.7	8.3 ± 4.6	0.063
Smoke tobacco	44 (14.7)	36 (17.1)	8 (9.0)	0.069
Drink alcohol	37 (12.4)	27 (12.9)	10 (11.2)	0.697
Exercise	51 (17.1)	34 (16.2)	17 (19.1)	0.541
Montreal Cognitive Assessment (adjusted)^a^	23.8 ± 5.6	22.8 ± 5.9	26.2 ± 3.8	< 0.001
Montreal Cognitive Assessment (adjusted)^a^ < 26	154 (51.5)	122 (58.1)	32 (36.0)	< 0.001
Charlson Comorbidity Index	1.1 ± 1.0	1.3 ± 1.1	0.9 ± 1.0	0.002
Charlson Comorbidity Index ≥2	90 (30.1)	74 (35.2)	16 (18.0)	0.003
Body mass index	25.3 ± 3.6	25.2 ± 3.8	25.3 ± 3.1	0.797

^a^One point added for education year12

### Subgroup comparisons

People with high versus low value-based health status were significantly younger, and cognitively intact (Fig. 1, Table 2); body mass index, alcohol consumption and exercise habits were similar between low versus high care status groups. Figure 1, Tables 1, and 3 summarize the proportions of 299 people in different ICHOM Tier categories, both overall and stratified by, value-based healthcare status (high vs low), age (< 75 vs ≥75 years), sex, and cognitive performance (MoCA_adj_ < 26 vs ≥26).

**Table 3:** Demographic data and ICHOM Standard Set Older Person outcome measures by sex, age, and cognitive performance status

Data values show mean ± standard deviation or number (percent)	Sex			Age (years)			MoCA_**adj**_		
	Female	Male	*p*	< 75	≥75	*p*	≥26	< 26	*p*
**Demographics and health-related factors**									
Number	177	122		184	115		145	154	
Age (years)	73.3 ± 6.3	73.3 ± 7.0	0.957	68.9 ± 2.7	80.3 ± 4.6	< 0.001	71.3 ± 5.5	75.1 ± 7.0	< 0.001
Male	0 (0)	122 (100)	< 0.001	75 (40.8)	47 (40.9)	0.985	57 (39.3)	65 (42.2)	0.61
Education (years)	6.9 ± 4.4	8.8 ± 4.8	0.001	8.3 ± 4.3	6.5 ± 5.0	0.001	9.3 ± 4.0	6.0 ± 4.7	< 0.001
Smoke tobacco	6 (3.4)	38 (31.2)	< 0.001	29 (15.8)	15 (13.0)	0.519	21 (14.5)	23 (14.9)	0.912
Drink alcohol	7 (4.0)	30 (24.6)	< 0.001	25 (13.6)	12 (10.4)	0.421	24 (16.6)	13 (8.4)	0.033
Exercise (≥30 min, ≥3 times/week)	31 (17.5)	20 (16.4)	0.8	37 (20.1)	14 (12.2)	0.076	24 (16.6)	27 (17.5)	0.822
Montreal Cognitive Assessment (adjusted)^a^	23.8 ± 5.5	23.7 ± 5.6	0.778	25.2 ± 4.6	21.5 ± 6.2	< 0.001	28.2 ± 1.6	19.5 ± 4.6	< 0.001
Montreal Cognitive Assessment (adjusted)^a^ < 26	89 (50.3)	65 (53.3)	0.61	75 (40.8)	79 (68.7)	< 0.001	0 (0.0)	154 (100.0)	< 0.001
Charlson Comorbidity Index	1.1 ± 1.1	1.2 ± 1.0	0.722	1.2 ± 1.0	1.1 ± 1.1	0.517	1.1 ± 1.0	1.2 ± 1.1	0.237
Charlson Comorbidity Index ≥2	48 (27.1)	42 (34.4)	0.176	60 (32.6)	30 (26.1)	0.232	42 (29.0)	48 (31.2)	0.678
Body mass index (kg/m^2^)	24.9 ± 3.7	25.8 ± 3.5	0.039	25.8 ± 3.4	24.4 ± 3.9	0.002	25.3 ± 3.3	25.2 ± 3.9	0.930
**ICHOM Standard Set Older Person Tier 1**									
Clinical frailty scale	2.6 ± 0.9	2.7 ± 1.0	0.838	2.5 ± 0.8	3.0 ± 1.0	< 0.001	2.5 ± 0.7	2.8 ± 1.1	< 0.001
Frail	22 (12.4)	13 (10.7)	0.639	13 (7.1)	22 (19.1)	0.002	5 (3.5)	30 (19.5)	< 0.001
Preferred place of death chosen	30 (17.0)	13 (10.7)	0.128	29 (15.8)	14 (12.2)	0.390	19 (13.1)	24 (15.6)	0.541
Do Not Resuscitate signed	15 (8.5)	6 (4.9)	0.237	17 (9.2)	4 (3.5)	0.058	15 (10.3)	6 (3.9)	0.029
**ICHOM Standard Set Older Person Tier 2**									
Number of drugs	3.4 ± 2.6	3.8 ± 2.7	0.207	3.2 ± 2.6	4.2 ± 2.7	0.001	3.5 ± 2.7	3.7 ± 2.7	0.436
Polypharmacy (≥5 concurrent drugs)	53 (29.9)	42 (34.4)	0.413	49 (26.6)	46 (40.0)	0.016	43 (29.7)	52 (33.8)	0.445
Number of adverse drug events	0.0 ± 0.2	0.0 ± 0.1	0.839	0.0 ± 0.2	0.0 ± 0.0	0.289	0.0 ± 0.2	0.0 ± 0.0	0.181
Episodes of discomfort after medications	0.0 ± 0.2	0.0 ± 0.1	0.599	0.0 ± 0.2	0.0 ± 0.0	0.103	0.0 ± 0.2	0.0 ± 0.0	0.103
Fell	34 (19.2)	17 (13.9)	0.233	27 (14.7)	24 (20.9)	0.166	24 (16.6)	27 (17.5)	0.822
Number of falls	0.3 ± 0.6	0.2 ± 0.5	0.131	0.2 ± 0.6	0.2 ± 0.5	0.672	0.2 ± 0.6	0.2 ± 0.6	0.877
Hospital admissions	0.1 ± 0.3	0.3 ± 0.6	< 0.001	0.2 ± 0.5	0.2 ± 0.4	0.871	0.2 ± 0.4	0.2 ± 0.5	0.657
Length of hospital stay (days)	0.5 ± 2.6	2.0 ± 4.8	0.003	1.0 ± 3.5	1.3 ± 4.1	0.486	0.9 ± 2.7	1.3 ± 4.5	0.286
Able to cope with own health	162 (91.5)	114 (93.4)	0.541	172 (93.5)	104 (90.4)	0.337	138 (95.2)	138 (89.6)	0.071
Participate in care decision-making	163 (92.1)	113 (92.6)	0.865	168 (91.3)	108 (93.9)	0.410	135 (93.1)	141 (91.6)	0.616
Treated with dignity and respect	170 (96.1)	119 (97.5)	0.48	178 (96.7)	111 (96.5)	0.919	142 (97.9)	147 (95.5)	0.234
Received coordinated care	163 (92.1)	107 (87.7)	0.208	159 (86.4)	111 (96.5)	0.004	132 (91.0)	138 (89.6)	0.678
Discharged to place of choice	176 (99.4)	120 (98.4)	0.36	183 (99.5)	113 (98.3)	0.313	144 (99.3)	152 (98.7)	0.597
Overall participation in decision-making	4.7 ± 0.8	4.7 ± 0.7	0.863	4.7 ± 0.8	4.8 ± 0.7	0.353	4.8 ± 0.6	4.6 ± 0.8	0.175
High participation (≥5 components)	150 (84.8)	94 (77.1)	0.091	147 (79.9)	97 (84.4)	0.333	122 (84.1)	122 (79.2)	0.273
**ICHOM Standard Set Older Person Tier 3**									
UCLA Loneliness Scale	30.7 ± 10.2	31.3 ± 9.8	0.611	30.4 ± 9.4	31.8 ± 11.0	0.232	28.4 ± 8.3	33.4 ± 11.0	< 0.001
Loneliness	46 (26.0)	34 (27.9)	0.718	46 (25.0)	34 (29.6)	0.386	22 (15.2)	58 (37.7)	< 0.001
Activities of daily living	7.5 ± 1.3	7.2 ± 1.5	0.08	7.6 ± 1.1	7.1 ± 1.7	0.002	7.8 ± 0.8	7.1 ± 1.8	< 0.001
Any limitation of activities of daily living	29 (16.4)	38 (31.2)	0.003	29 (15.8)	38 (33.0)	< 0.001	19 (13.1)	48 (31.2)	< 0.001
Walking speed (m/s)	0.9 ± 0.3	0.9 ± 0.3	0.514	0.9 ± 0.3	0.8 ± 0.3	< 0.001	1.0 ± 0.2	0.8 ± 0.3	< 0.001
Slowness (6-m walk < 0.8 m/s)	68 (38.4)	47 (38.5)	0.985	54 (29.4)	61 (53.0)	< 0.001	32 (22.1)	83 (53.9)	< 0.001
Moderate pain	21 (11.9)	10 (8.2)	0.307	15 (8.2)	16 (13.9)	0.112	16 (11.0)	15 (9.7)	0.714
Depression	25 (14.1)	14 (11.5)	0.504	19 (10.3)	20 (17.4)	0.078	16 (11.0)	23 (14.9)	0.317
Value-based health score	7.3 ± 1.9	7.1 ± 1.8	0.312	7.6 ± 1.6	6.7 ± 2.1	< 0.001	7.8 ± 1.5	6.8 ± 2.0	< 0.001
High value-based health	60 (33.9)	29 (23.8)	0.06	63 (34.2)	26 (22.6)	0.032	57 (39.3)	32 (20.8)	0.001

^a^One point added for education years ≤12

*ICHOM* International Consortium for Health Outcomes Measurement, *UCLA* University of California, Los Angeles

### Linear and logistic regression analyses

Younger age, lower Charlson Comorbidity Index score, and higher MoCA_adj_ score independently predicted high value-based healthcare status (Table 4). For every year increase in age, the likelihood of achieving high value-based healthcare status decreased by 5%. (Table 5). People with higher disease burden and cognitive impairment were 60 and 51% less likely, respectively, to attain high status (Table 5).

**Table 4:** Factors associated with high-value health status in univariable and multivariate linear regression analyses

	Univariable		Multivariable			
	β coefficient	*p*	β coefficient^a^	*p*	β coefficient^b^	*p*
Age (years)	−0.096	< 0.001	−0.050	0.001	−0.079	< 0.001
Male	−0.196	0.364				
Education (years)	0.080	< 0.001	0.002	0.921	0.035	0.110
Smoke tobacco	−0.480	0.105				
Drink alcohol	0.034	0.917				
Exercise	0.340	0.231	0.170	0.488	0.161	0.525
CCI	−0.366	< 0.001	−0.297	0.001		
CCI ≥2	−0.845	< 0.001			−0.832	< 0.001
MoCA_adj_	0.155	< 0.001	0.129	< 0.001		
MoCA_adj_ < 26	−0.998	< 0.001			−0.591	0.005
Body Mass Index	0.058	0.048	0.038	0.135	0.032	0.237

*CCI* Charlson Comorbidity Index, *MoCA*_*adj*_ Montreal Cognitive Assessment adjusted (one point added for education years ≤12)

^a^CCI and MoCA_adj_ as numerical variables

^b^CCI and MoCA_adj_ as categorical variables

**Table 5:** Factors associated with high-value health status in univariable and multivariable logistic regression analyses

	Univariable		Multivariable			
	Odds ratio (95% CI)	*p*	Odds ratio (95% CI)^a^	*p*	Odds ratio (95% CI)^b^	*p*
Age (years)	0.94 (0.90, 0.98)	0.003	0.97 (0.92, 1.02)	0.176	0.95 (0.91, 0.99)	0.025
Male	0.61 (0.36, 1.02)	0.061	0.69 (0.37,1.28)	0.241	0.70 (0.38, 1.29)	0.257
Education (years)	1.05 (0.99, 1.11)	0.092	0.99 (0.93, 1.06)	0.783	1.02 (0.96, 1.09)	0.593
Smoke tobacco	0.48 (0.21, 1.07)	0.074	0.57 (0.23, 1.42)	0.228	0.53 (0.21, 1.32)	0.172
Drink alcohol	0.86 (0.40, 1.86)	0.697				
Exercise	1.22 (0.64, 2.33)	0.541				
CCI	0.65 (0.49, 0.85)	0.002	0.66 (0.49, 0.89)	0.006		
CCI ≥2	0.40 (0.22, 0.74)	0.004			0.40 (0.21, 0.76)	0.005
MoCA_adj_	1.15 (1.08, 1.22)	< 0.001	1.15 (1.07, 1.23)	< 0.001		
MoCA_adj_ < 26	0.41 (0.24, 0.68)	0.001			0.49 (0.27, 0.87)	0.014
Body Mass Index	1.01 (0.94, 1.08)	0.813				

*CI* confidence interval, *CCI* Charlson comorbidity index, *MoCA*_*adj*_ Montreal Cognitive Assessment adjusted (one point added for education years ≤12)

^a^CCI and MoCA_adj_ as numerical variables

^b^CCI and MoCA_adj_ as categorical variables

## Discussion

This is the first study of which we know to report the value-based healthcare status of older multimorbid community-living adults. We applied the ICHOM Standard Set for Older Person to evaluate the value-based healthcare status of ≥65-year-olds with multimorbidity; those who were younger, and cognitively unimpaired had higher levels of value-based healthcare status. Rates of participation in decision-making were high across all subgroups. Having high disease burden and impaired cognitive function were negatively associated with ability to achieve a high value-based healthcare score.

ICHOM Standard Set Older Person categorization into three tiers is based on Porter’s health outcome hierarchy [17]. Tier 1 includes peoples’ preferences for end-of-life care; choosing a place of death helps people to die at home, whereas people whose preference is unknown are more likely to be admitted to hospital for end-of-life care [18]. The proportion of Asian participants in this study expressing a preferred place of death or signing a Do Not Recuscitate Agreement was low compared with other studies [19]; this highlights an unmet need for advocacy to better prepare elderly Taiwanese people for death. The prevalence of frailty was similar to other reports [8, 20].

Approximately one-third of participants used ≥5 concurrent medications, consistent with a study of national health insurance claims by 59,042 Taiwanese people older than 65 years [21]; nevertheless there was a low incidence of adverse drug events or discomfort after taking medications, likely due to a low rate of inappropriate medication according to insurance claims data [21]. Although people prefer more participation in decision-making and expect to be treated with dignity and respect, not all patients want to make medical decisions [22]. More than 90% of people in our study participated in decisions about their care- and received collaborative, dignified and respectful medical management, compared with 60% in a systemic review of 44 studies [22]. Although falling is usually considered a health outcome, it was chosen as a standard value-based metric because it matters to older people, their carers, and physicians. A higher rate of falls among women than men in this study was consistent with a study of 1377 community-living Taiwanese, although not statistically significant [23].

The use of SF-36 in Tier 3 to measure depression and pain has the advantage of covering many outcomes to reduce complexity, but some experts advocate considering cost-free survey tools as well [5]. The prevalence of depression in this study population was similar to previous reports from dermatology and internal medicine, and lower than among surgery patients [24]. A meta-analysis study of 19 studies reported moderate to severe chronic pain in 10–14% participants [25], which was similar to our findings. Participation and social inclusion are key components of healthy aging; 26.8% prevalence of loneliness was consistent with previous reports [26]. Although measuring physical performance is not always easy in daily practice, the ICHOM included walk speed as a Tier 3 metric because it matters to older adults [27]; the mean speed of 0.9 m/s in this sample was much lower than reported in older healthy adults [28], reflecting that all participants were multimorbid.

The ICHOM Standard Set of outcome measures was the first tool developed for people who are older, rather than those with specific diseases or conditions. Based on findings from the study, stakeholders may devise tailor-made interventions for this population and examine their effectiveness accordingly. However, the ICHOM Standard Set does not include cognitive assessment; our results show that cognitive function per se was highly associated with high value-based healthcare status and might be considered amended in metrics of the ICHOM Standard Set. The ICHOM variables are a combination of self-reported and professional-assessed. Most of these variables are not routinely collected, even in an integrated geriatric health care clinic. That might burden collection of data from patients and professionals. For example, a Chinese version of the 4-item screening Zarit Burden interview was not available at the time this study commenced. Complexity and burden of assessments might be a barrier to scale up use of the standard set in Taiwan or other countries, Akpan et al. argued that free tools that encompass multiple outcomes to reduce numbers of measurable variables and complexity of tools would be help for clinical and public health implementation [5].

This study had limitations. First, the ICHOM Standard Set was designed to measure longitudinal changes of value-based health status; our cross-sectional study only presents a snapshot of baseline status. Second, convenient sampling instead of random sampling limits the representativeness and generalizability, although various dimensions studied had profiles similar to previous studies. Extrapolation of our study results to other populations may need further validation. Third, questionnaire items about falls and drug adverse events over the past year may result in recall-bias; this could be resolved by a prospective study, which is underway, and we intend to report in due course.

## Conclusions

ICHOM Standard Set health outcome measures provide an opportunity to shift from a disease-centric medical paradigm to whole person care goals. The value-based health care profile in Taiwan indicates the importance of advanced age, chronic disease and cognitive impairment as barriers to achieving high value-based health status. Further longitudinal and intervention studies to examine the expedience of using ICHOM are warranted.

## Supplementary information

**Additional file 1: Supplementary Table 1.** Value-health points score components by ICHOM Standard Set outcome measures

**Additional file 2: Supplementary figure 1.** Flow diagram of participants recruiting process in the study.

## Data Availability

The datasets generated and/or analysed during the current study are not publicly available due local government regulations but are available from the corresponding author on reasonable request.

## Abbreviations

ICHOM: International Consortium for Health Outcomes Measurement
UCLA: University of California, Los Angeles
SF-36: Short-Form Health Survey
MoCA,: Montreal Cognitive Assessment
MoCAadj: Montreal Cognitive Assessment adjusted by adding one point for those educated for ≤12 years
CCI: Charlson Comorbidity Index
