# Supplementary material for: First insights on value-based healthcare of elders using ICHOM older person standard set reporting
Source: BMC Geriatr. 2020 Sep 9;20:335. doi: 10.1186/s12877-020-01734-1 (PMC7487791; doi:10.1186/s12877-020-01734-1)
Supplement: Supplementary file 1 — Additional file 1: Supplementary Table 1. Value-health points score components by ICHOM Standard Set outcome measures [file 12877_2020_1734_MOESM1_ESM.docx]

| **Supplementary Table 1** Value-health points score components by ICHOM Standard Set outcome measures | | | | | | | | | |
| --- | --- | --- | --- | --- | --- | --- | --- | --- | --- |
| **ICHOM Tiers** | | **ICHOM outcome domains** | | **ICHOM outcome domain component metrics** | | **Value-health score** | | | |
|  |  |  |  |  |  | 0 point | | 1 point | |
| **Tier 1** | Place of death | | Reply yes or no to "whether a preferred place of death has been expressed?" | | No | | Yes | |  |
|  | Frailty | | Clinical frailty scale | | ≥4 | | <4 | |  |
| **Tier 2** | | Polypharmacy | | Number of drugs for chronic conditions | | ≥5 | | <5 | |
|  |  | Falls | | Any fall in the past 12 months | | Yes | | No | |
|  |  | Participation in decision making | | 1) Confident in ability to manage their own health; 2) Participate in discussion and planning of care: 3) Treated with dignity and respect; 4) Coordinated care; 5) Discharge to chosen place. | | No to any | | Yes to 5/5 | |
| **Tier 3** | | Loneliness and isolation | | UCLA 3-item scale | | ≥35 | | <35 | |
|  |  | Limited activities of daily living | | Any limitation of Lawton instrumental activities of daily living scale | | Yes | | No | |
|  |  | Slowness | | 6-metre walk speed | | <0.8 m/s | | ≥0.8 m/s | |
|  |  | Pain | | Moderate pain affecting activities of daily living | | Yes | | No | |
|  |  | Depressed mood and emotional ill-health | | ≥5/9 emotional health symptoms measured by the Short-Form Health Survey | | Yes | | No | |
| ICHOM, International Consortium for Health Outcomes Measurement; UCLA, University of California, Los Angeles. | | | | | | | | | |
