# Supplementary figures and images for: First insights on value-based healthcare of elders using ICHOM older person standard set reporting
Source: BMC Geriatr. 2020 Sep 9;20:335. doi: 10.1186/s12877-020-01734-1 (PMC7487791; doi:10.1186/s12877-020-01734-1)

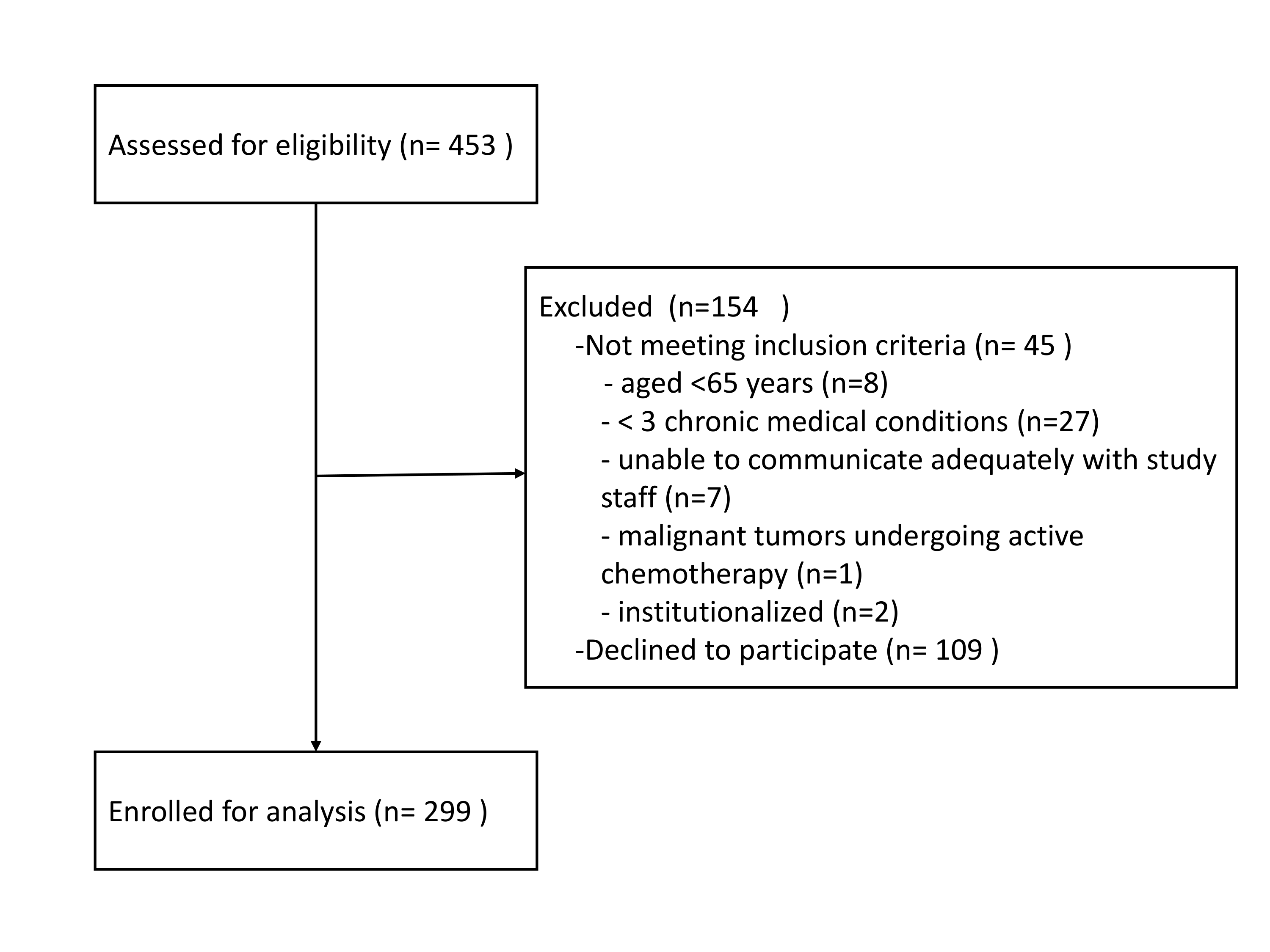

Supplement: Supplementary file 2 — Additional file 2: Supplementary figure 1. Flow diagram of participants recruiting process in the study. [file 12877_2020_1734_MOESM2_ESM.tif]
